# Oxidative Stress-Induced Adverse Effects of Three Statins Following Single or Repetitive Treatments in Mice

**DOI:** 10.7759/cureus.51433

**Published:** 2024-01-01

**Authors:** Rawnaq F Al-Shalchi, Fouad K Mohammad

**Affiliations:** 1 Department of Physiology, Biochemistry and Pharmacology, College of Veterinary Medicine, University of Mosul, Mosul, IRQ; 2 College of Nursing, The American University of Kurdistan, Duhok, IRQ

**Keywords:** hypolipidemic drugs, brain injury, liver injury, liver enzymes, glutathione, malondialdehyde, rosuvastatin, simvastatin, atorvastatin

## Abstract

Background and objective

The hypolipidemic statins have been associated with various side effects, and in some cases, adverse reactions in humans and experimental animals, such as myotoxicity, neurobehavioral toxicity, as well as liver and kidney injuries. The purpose of the present study was to examine the possibility of the induction of oxidative stress in the brain and plasma of mice dosed with single or repetitive doses of three statins (atorvastatin, simvastatin, and rosuvastatin).

Methods

Male Swiss-origin mice were dosed orally with single doses of each of the three statins at 500 or 1000 mg/kg of body weight. Other groups of mice were dosed orally with repeated daily doses of each of the statins at 200 mg/kg of body weight/day for 14 or 28 consecutive days. These doses of statins were chosen to not produce overt toxicity in mice within the time frame allocated for each experiment. Brain and plasma glutathione (GSH) and malondialdehyde (MDA) levels, as well as liver enzymes activities alanine transaminase (ALT) and aspartate transaminase (AST), were determined using commercial kits.

Results

Single-dose treatments of the mice with the statins at either 500 or 1000 mg/kg significantly and dose-dependently (p < 0.05) reduced the GSH level in the plasma and the whole brain when compared with respective control values. Atorvastatin was the least effective statin, as only the high dose achieved a significant reduction in brain GSH level in comparison with the respective control value. Repetitive administration of the three statins at 200 mg/kg of body weight/day for 14 or 28 consecutive days significantly and time-dependently reduced plasma and brain GSH levels in comparison with respective control values. The oxidative stress biomarker MDA level significantly increased in the plasma and brain of mice following single or repetitive treatments with the three statins, and the most effective one was rosuvastatin. In association with these changes, activities of the liver enzymes ALT and AST were also increased in the plasma with single and repetitive statin treatments, and the most effective one was rosuvastatin.

Conclusion

The data suggest an association of high doses of three statins (atorvastatin, simvastatin, and rosuvastatin) with the induction of oxidative stress manifested as GSH reduction and MDA elevation as adverse effects in the brain and plasma of mice, which suffered from the additional burden of liver injury. These effects could be the basis of an in-depth exploration of statin adverse effects in experimental animals and to find an animal model, probably the mice, for the induction of adverse effects of statins that target the brain, as well as to shed light on potential statin intolerance outcomes following single-dose treatments in this species.

## Introduction

Many statins with varying structures and chemical and physical properties are clinically used to treat hypercholesterolemia by blocking the synthesis of cholesterol in the liver following the inhibition of the rate-limiting enzyme hydroxyl-methyl-glutaryl-CoA reductase [1,2]. Statins are characterized by a wide margin of safety; however, side effects or even adverse outcomes in association with acute and chronic treatment regimens have been reported clinically [1,3-5]. These adverse effects include but are not limited to myotoxicity, neurotoxicity, liver and kidney toxicity, as well as many biochemical alterations [3-7]. Many studies in experimental animals have also supported and documented adverse effects of statins, such as neurobehavioral and locomotive changes [8-11], impairment of neuromuscular function [12], and alterations in the cholinergic system [10,13]. More recently, we have found adverse effects of statins to be associated with diverse neurobehavioral changes and reduced brain and blood cholinesterase activity in mice [14]. Within this context, examining statins' toxicity or adverse effects became warranted in light of the withdrawal of one statin from clinical use [15] and the actual clinical or perceived statin adverse effects [1,3-5], as well as statin intolerance among certain hyperlipidemic patients [16,17].

Various mechanistic investigations have indicated the involvement of several exacerbated biochemical events in the induction of statin intolerance, adverse effects, and toxicity. For example, adverse/toxic outcomes of statins may include reduced blood or tissue cholinesterase activities [10,13,14], hyperkalemia [18], impaired mitochondrial function [19], and generation of reactive oxygen species causing oxidative stress [20]. Hepatocellular liver injury with concomitant elevations of alanine transaminase (ALT), aspartate transaminase (AST), and alkaline phosphatase have been seen during various therapeutic applications of statins irrespective of the duration of therapy [21]. However, it has been argued that the therapeutic benefits of statins could be related to their antioxidant actions, which are also implicated in the pleiotropic effects of these drugs [22,23]. In light of the complex features of statins' effects, whether therapeutic or adverse outcomes, with regards to their prooxidant-antioxidant impacts [20-23], the present study was undertaken in mice to examine adverse effects of three commonly used statins (atorvastatin, simvastatin, and rosuvastatin) on the levels of brain and plasma malondialdehyde (MDA), a metabolic biomarker of oxidative stress, and the antioxidant glutathione (GSH). Liver enzymes ALT and AST were also determined in the plasma of the statin-treated mice.

## Materials and methods

Animals and ethics

In this experimental study, we used male Swiss-origin adult mice (age 100-120 days) with body weights ranging from 30 to 35 g. Animal housing conditions were standardized at temperatures between 20 to 24 ºC and a 12-h light/dark cycle, with free access to water and laboratory rodent food. This research project was reviewed and approved by the Departmental Scientific Committee on Research and Animal Care and Use. We also obtained the approval of the Committee of Postgraduate Studies at the College of Veterinary Medicine, University of Mosul, Iraq, to conduct the study in mice according to the institutional regulations and ethics on the use of laboratory animals and their handling in biomedical research, in compliance with the guidelines of Animal Research: Reporting of In Vivo Experiments (ARRIVE) (https://www.nc3rs.org.uk/arrive-guidelines) and the Guide for the Care and Use of Laboratory Animals [24]. The Institutional Review Board approval was granted by the College of Veterinary Medicine, University of Mosul (No. 2144, November 2, 2022), after the University of Mosul provided permission (No. 4S/29927, October 30, 2022). Human participants or tissues were not used in the present study.

Drugs used

**Figure 1:**
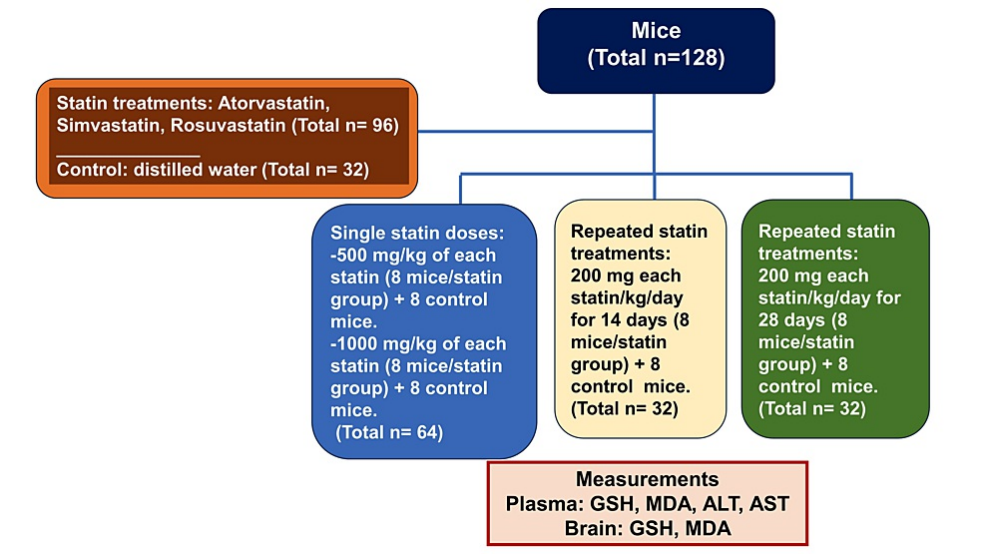
The integrated experimental design of the study and allocation of mice to single or repeated doses of three statins, while distilled water treatment groups were used as controls. n: numbers of mice used within each treatment regimen or control group; GSH: glutathione; MDA: malondialdehyde; ALT: Alanine transaminase; AST: aspartate transaminase.

The statins used, atorvastatin, simvastatin, and rosuvastatin, were kindly donated by the State Company for Drugs Industry and Medical Appliances, Samarra, Iraq. The required drug concentrations were freshly prepared in distilled water as a vehicle for oral dosing by a gavage needle at a volume of 10 mL/kg of body weight. Figure 1 outlines the experimental protocol and allocation of 128 mice to different statin treatment regimens (n= 96) and their appropriate control counterparts treated with distilled water (n= 32). The doses and treatment regimens of the three statins were as follows: Single-dose treatments consisted of oral dosing of eight mice per group with each of the three statins at 500 or 1000 mg/kg of body weight. Eight control mice were treated with distilled water concurrently with each statin dose level. Repeated doses with each of the statins were at 200 mg/kg of body weight/day for 14 or 28 consecutive days (8 mice/dose group of each statin/14 days or /28 days), and two control groups of eight mice each were used for each time period. According to a previous study [14] and our own preliminary experiments, these doses of statins did not produce overt toxicity in mice within the time frame allocated for each experiment. The mice were randomly allocated to treatment groups.

Samples and measurements

Blood samples were withdrawn from the retro-orbital plexus under terminal anesthesia with ether into heparinized capillary tubes two hours after the single-dose treatments with the three statins or with distilled water (control), as well as 24 hours after repetitive statin or distilled water (control) dosing for 14 and 28 days. Thereafter, blood samples were centrifuged at 3000 rpm for 15 minutes to separate the plasma. The whole brain was dissected out and homogenized with normal saline (1:9) using a homogenizer (OMNI Bead Ruptor, OMNI International, United States) at a speed of 400 rounds/second.

Brain and plasma GSH and MDA levels were determined by commercial kits purchased from Elabscience Biotechnology Inc., Houston, Texas, United States. Plasma ALT and AST were determined using commercial kits obtained from BIOLABO S.A.S., Les Hautes Rives 02160 Maizy, France.

Statistics

The data were statistically analyzed by analysis of variance followed by the least significant difference test, using IBM SPSS Statistics for Windows, Version 20 (Released 2011; IBM Corp., Armonk, New York). The level of statistical significance was at p < 0.05.

## Results

Single-dose statin treatments

Single-dose treatments with the statins atorvastatin, simvastatin, and rosuvastatin at 500 and 1000 mg/kg of body weight significantly (p < 0.05) and dose-dependently reduced GSH levels in the plasma and the whole brain when compared with respective control values (Table 1). The most effective statin in causing significant reductions in plasma and brain GSH levels was rosuvastatin at 1000 mg/kg (mean ± SE, 36.533 ± 1.33 mg/L and 0.742 ± 0.03 mg/g protein, respectively) when compared with respective control values (69.7 ± 1.43 and 1.411 ± 0.02), and in comparison with the reductions caused by atorvastatin (56.49 ± 1.17 and 1.137 ± 0.03) and simvastatin (50.303 ± 1.07 and 1.087 ± 0.03). Atorvastatin had the least effect on GSH since only its high dose achieved a significant reduction in brain GSH level (1.137 ± 0.03) in comparison with the respective control value (1.411 ± 0.02).

**Table 1:** Plasma and brain glutathione levels in mice two hours after a single dose oral treatment with statins Values are mean ± SE of 8 mice/group. GSH = glutathione. ^*^p-value for the statistical difference from the respective control group. ^a^p-value for the statistical difference from the respective atorvastatin dose group. ^b^p-value for the statistical difference from the respective simvastatin dose group. ^†^p-value for the statistical difference from the respective 500 mg/kg dose group of the same statin. Analysis of variance: plasma GSH data, F= 75.038; brain GSH data, F= 61.319, p < 0.0001. Statistically significant difference was set at p < 0.05.

Statin groups	Single statin dose			
	500 mg/kg	p-value	1000 mg/kg	p-value
Plasma GSH (mg/L)				
Distilled water control	65.707 ± 1.66	-	69.700 ± 1.43	0.03^†^
Atorvastatin	54.131 ± 1.48	0.0001^*^	56.490 ± 1.17	0.0001^*^, 0. 192^†^
Simvastatin	48.930 ± 1.00	0.0001^*^, 0.005^a^	50.303 ± 1.07	0.0001^*^, 0.001^a^, 0.445^†^
Rosuvastatin	43.496 ± 0.70	0.0001^*a^, 0.0004^b^	36.533 ± 1.33	0.0001^*ab†^
Brain GSH (mg/g protein)				
Distilled water	1.405 ± 0.03	-	1.411 ± 0.02	0.874^†^
Atorvastatin	1.327 ± 0.02	0.055^*^	1.137 ± 0.03	0.0001^*†^
Simvastatin	1.182 ± 0.02	0.0001^*a^	1.087 ± 0.03	0.0001^*^, 0.208^a^, 0.019^†^
Rosuvastatin	1.127 ± 0.03	0.0001^*a^, 0.166^b^	0.742 ± 0.03	0.0001^*ab†^

The two single doses of each statin (500 and 1000 mg/kg) significantly increased the level of the oxidative stress biomarker MDA in the plasma and brain of mice in a dose-dependent manner in comparison with respective control values (Table 2). The statin that caused the highest significant increase in MDA level in the plasma (983.4 ± 8.46 ng/mL) and whole brain (948.225 ± 5.17 ng/mg) was rosuvastatin at 1000 mg/kg versus respective control values (75.916 ± 2.0 and 76.012 ± 2.09, respectively). These plasma and brain MDA levels in rosuvastatin-treated mice were even significantly higher than those of the respective atorvastatin (180.842 ± 3.69 and 172.875 ± 2.35) and simvastatin (573.780 ± 5.19 and 536.737 ± 5.19) groups. Single doses of atorvastatin (500 and 1000 mg/kg) had the least effect on MDA level in the plasma (159.250 ± 3.17 and 180.842 ± 3.69) and brain (133.0 ± 1.81 and 172.875 ± 2.35) of mice versus respective plasma (72.075 ± 2.40 and 75.916 ± 2.0) and brain (69.167 ± 2.64 and 76.012 ± 2.09) control values.

**Table 2:** Plasma and brain malondialdehyde levels in mice two hours after a single dose oral treatment with statins Values are mean ± SE of 8 mice/group. MDA = malondialdehyde. ^*^p-value for the statistical difference from the respective control group. ^a^p-value for the statistical difference from the respective atorvastatin dose group. ^b^p-value for the statistical difference from the respective simvastatin dose group. ^†^p-value for the statistical difference from the respective 500 mg/kg dose group of the same statin. Analysis of variance: plasma MDA data, F = 5368.996; brain MDA data, F = 8076.438, p < 0.0001. Statistically significant difference was set at p < 0.05.

Statin groups	Single statin dose			
	500 mg/kg	p-value	1000 mg/kg	p-value
Plasma MDA (ng/mL)				
Distilled water	72.075 ± 2.40	-	75.916 ± 2.00	0.548^†^
Atorvastatin	159.250 ± 3.17	0.0001^*^	180.842 ± 3.69	0.0001^*†^
Simvastatin	226.312 ± 2.63	0.0001^*a^	573.780 ± 5.19	0.0001^*a†^
Rosuvastatin	655.087 ± 4.80	0.0001^*ab^	983.400 ± 8.46	0.0001^*ab†^
Brain MDA (ng/mg)				
Distilled water	69.167 ± 2.64	-	76.012 ± 2.09	0.184^†^
Atorvastatin	133.00 ± 1.81	0.0001^*^	172.875 ± 2.35	0.0001^*†^
Simvastatin	222.812 ± 1.86	0.0001^*a^	536.737 ± 5.19	0.0001^*a†^
Rosuvastatin	652.875 ± 5.15	0.0001^*ab^	948.225 ± 5.17	0.0001^*ab†^

Concomitantly with single-dose changes in GSH and MDA levels, the liver enzyme ALT in the plasma of mice treated with single doses of the statins (500 and 1000 mg/kg) were significantly above the respective control values (Table 3). On the other hand, at 500 mg/kg, only rosuvastatin significantly increased plasma AST activity in comparison with the respective control value, whereas atorvastatin and simvastatin effects did not attain statistically significant differences (p > 0.05). However, the three statins showed a dose-dependent effect, as the 1000 mg/kg dose level significantly increased plasma AST activity in comparison with the control value (Table 3). Additional statistical analysis revealed that the most prominent and significant adverse effects on ALT and AST activities were due to rosuvastatin treatments at 500 mg/kg (27.157 ± 0.51 and 37.446 ± 1.11 IU/L, respectively) and 1000 mg/kg (30.842 ± 0.62 and 53.661 ± 1.08, respectively) when compared to respective plasma ALT (4.568 ± 0.21 and 4.802 ± 0.23) and AST (10.815 ± 0.56 and 10.701 ± 0.47) control values.

**Table 3:** Plasma alanine transaminase and aspartate transaminase activities in mice two hours after a single dose oral treatment with statins Values are mean ± SE of 8 mice/group. ALT = alanine transaminase; AST= aspartate transaminase. ^*^p-value for the statistical difference from the respective control group. ^a^p-value for the statistical difference from the respective atorvastatin dose group. ^b^p-value for the statistical difference from the respective simvastatin dose group. ^†^p-value for the statistical difference from the respective 500 mg/kg dose group of the same statin. Analysis of variance: ALT data, F = 245.973; AST data, F = 364.383, p < 0.0001. Statistically significant difference was set at p < 0.05.

Statin groups	Single statin dose			
	500 mg/kg	p-value	1000 mg/kg	p-value
Plasma ALT (IU/L)				
Distilled water control	4.568 ± 0.21	-	4.802 ± 0.23	0.233†
Atorvastatin	12.187 ± 1.33	0.0001^*^	12.846 ± 0.41	0.0001^*^
Simvastatin	11.516 ± 0.42	0.0001^*^ 0.439^a^	16.003 ± 0.32	0.0001^*a†^
Rosuvastatin	27.157 ± 0.51	0.0001^*ab^	30.842 ± 0.62	0.0001^*†ab^
Plasma AST (IU/L)				
Distilled water	10.815 ± 0.56	-	10.701 ± 0.47	0.978^† ^
Atorvastatin	16.702 ± 0.48	0.153^*^	33.123 ± 0.90	0.0001^*†^
Simvastatin	17.096 ± 0.59	0.128^*^ 0.923^a^	25.240 ± 0.64	0.001^*^, 0.058^ a^, 0.05^†^
Rosuvastatin	37.446 ± 1.11	0.0001^*ab^	53.661 ± 1.08	0.0001^*†ab^

Repeated statin treatments

Repeated dosing of mice with each of the three statins at 200 mg/kg/day for 14- and 28 consecutive days significantly and time-dependently reduced plasma and brain GSH levels in comparison with respective control values (Table 4). Within these time frame treatment regimens, rosuvastatin had the most prominent effect among statin treatment groups on reducing plasma (27.402 ± 0.96 and 19.908 ± 0.85) and brain (0.747 ± 0.03 and 0.408 ± 0.03) GSH levels, respectively, when compared with concurrent control values in the plasma (67.333 ± 1.04 and 68.070 ± 1.10) and the brain (1.417 ± 0.02 and 1.442 ± 0.03).

**Table 4:** Plasma and brain glutathione levels in mice dosed orally with statins at 200 mg/kg of body weight/day for 14 or 28 consecutive days Values are mean ± SE of 8 mice/group. The mice were sacrificed 24 hours after the last 14- or 28-day consecutive treatments. GSH = glutathione. ^*^p-value for the statistical difference from the respective control group. ^a^p-value for the statistical difference from the respective atorvastatin dose group. ^b^p-value for the statistical difference from the respective simvastatin dose group. ^†^p-value for the statistical difference from the respective 14-day value of the same statin. Analysis of variance: plasma GSH data, F= 254.242; brain GSH, F= 50.059, p < 0.0001. Statistically significant difference was set at p < 0.05.

Statin groups	Duration of statin treatment			
	14 days	p-value	28 days	p-value
Plasma GSH (mg/L)				
Distilled water control	67.333 ± 1.04	-	68.070 ± 1.10	0.626^†^
Atorvastatin	45.572 ± 1.18	0.0001^*^	51.756 ± 1.10	0.0001^*†^
Simvastatin	44.773 ± 1.21	0.0001^*^ 0.597^a^	43.837 ± 1.00	0.0001^*a^, 0.536^†^
Rosuvastatin	27.402 ± 0.96	0.0001^*ab^	19.908 ± 0.85	0.0001^*†ab^
Brain GSH (mg/g protein)				
Distilled water	1.417 ± 0.02	-	1.442 ± 0.03	0.731^†^
Atorvastatin	1.122 ± 0.03	0.0001^*^	0.857 ± 0.03	0.0001^*^, 0.001^†^
Simvastatin	0.843 ± 0.12	0.0001^*a^	0.678 ± 0.03^*†a^	0.0001^*^, 0.017^a^, 0.0260^†^
Rosuvastatin	0.747 ± 0.03	0.0001^*a^, 0.189^b^	0.408 ± 0.03	0.0001^*†ab^

Similar to single-dose treatment regimens, the three statins after repetitive treatments for 14- and 28 consecutive days significantly and time-dependently increased plasma and brain MDA levels in comparison with respective control values (Table 5). The effects of rosuvastatin treatments for 14- and 28 days on MDA levels in the plasma (1134.153 ± 8.08 and 1234.221) and brain (566.712 ± 5.95 and 1082.00 ± 3.35) of mice were the most prominent and significant ones among the statins when compared with respective control values in the plasma (75.195 ± 1.64 and 81.812 ± 1.73) and the brain (64.190 ± 1.27 and 67.912 ± 2.22).

**Table 5:** Plasma and brain malondialdehyde levels in mice dosed orally with statins at 200 mg/kg of body weight/day for 14 or 28 consecutive days Values are mean ± SE of 8 mice/group. The mice were sacrificed 24 hours after the last 14- or 28-day consecutive treatments. MDA = malondialdehyde. ^*^p-value for the statistical difference from the respective control group. ^a^p-value for the statistical difference from the respective atorvastatin dose group. ^b^p-value for the statistical difference from the respective simvastatin dose group. ^†^p-value for the statistical difference from the respective 14-day value of the same statin. Analysis of variance: plasma MDA data, F= 8004.814; brain MDA, F= 10270.293, p < 0.0001. Statistically significant difference was set at p < 0.05.

Statin groups	Duration of statin treatment			
	14 days	p-value	28 days	p-value
Plasma MDA (ng/mL)				
Distilled water	75.195 ± 1.64	-	81.812 ± 1.73	0.378^†^
Atorvastatin	187.953 ± 4.25	0.0001^*^	187.568 ± 2.46	0.0001^*^, 0.959^†^
Simvastatin	268.850 ± 3.74	0.0001^*a^	620.487 ± 7.59	0.0001^*a†^
Rosuvastatin	1134.153 ± 8.08	0.0001^*ab^	1234.221 ± 7.42	0.0001^*†ab^
Brain MDA (ng/mg)				
Distilled water	64.190 ± 1.27	0.747	67.912 ± 2.22	0.747^†^
Atorvastatin	134.00 ± 1.77	0.0001^*^	226.037 ± 4.37	0.0001^*†^
Simvastatin	185.475 ± 2.20	0.0001^*a^	332.887 ± 3.51	0.0001^*a†^
Rosuvastatin	566.712 ± 5.95	0.0001^*ab^	1082.00 ± 3.35	0.0001^*ab†^

Repetitive administration of the three statins at 200 mg/kg/day for 14 or 28 consecutive days caused liver injury similar to that of single-dose statin treatments mentioned above. This was evident by significant and time-dependent elevations in plasma ALT and AST activities in comparison with respective control values (Table 6). The most injurious statin was rosuvastatin repetitive dosing for 14 and 28 consecutive days, as the ALT (44.065 ± 1.15 and 55.317 ± 1.22) and AST (56.036 ± 1.10 and 71.065 ± 1.35) activities were significantly elevated compared to those of respective control ALT (4.720 ± 0.20 and 4.638 ± 0.25) and AST (11.522 ± 0.49 and 13.037 ± 0.50) values.

**Table 6:** Plasma alanine transaminase and aspartate transaminase activities in mice dosed orally with statins at 200 mg/kg of body weight/day for 14 or 28 consecutive days Values are mean ± SE of 8 mice/group. The mice were sacrificed 24 hours after the last 14- or 28-day consecutive treatments. ALT = alanine transaminase; AST = aspartate transaminase. ^*^p-value for the statistical difference from the respective control group. ^a^p-value for the statistical difference from the respective atorvastatin dose group. ^b^p-value for the statistical difference from the respective simvastatin dose group. ^†^p-value for the statistical difference from the respective 14-day value of the same statin. Analysis of variance: ALT data, F= 728.139; AST data, F= 577.321, p < 0.0001. Statistically significant difference was set at p < 0.05.

Statin groups	Duration of statin treatment				
	14 days	p-value		28 days	p-value
Plasma ALT (IU/L)					
Distilled water	4.720 ± 0.20	-		4.638 ± 0.25	0.933^†^
Atorvastatin	12.682 ± 0.40	0.0001^*^		16.685 ± 0.38	0.0001^*†^
Simvastatin	16.670 ± 0.59	0.0001^*a^		21.311 ± 0.30	0.0001^*a†^
Rosuvastatin	44.065 ± 1.15	0.0001^*ab^		55.317 ± 1.22	0.0001^*ab†^
Plasma AST (IU/L)					
Distilled water	11.522 ± 0.49		-	13.037 ± 0.50	0.225^†^
Atorvastatin	21.225 ± 0.68		0.001^*^	26.338 ± 0.75	0.0001^*†^
Simvastatin	25.581 ± 0.48		0.0001^*^, 0.001^a^	28.971 ± 1.14	0.0001^*^, 0.037^a^, 0.008^†^
Rosuvastatin	56.036 ± 1.10		0.0001^*ab^	71.065 ± 1.35	0.0001^*†ab ^

## Discussion

The present study is an experimental mouse model we used to assess the oxidative stress induced by three statins (atorvastatin, simvastatin, and rosuvastatin) that differ in their pharmacokinetic and pharmacodynamic effects [1,2,25]. The three statins treatment regimens we applied in mice, consisting of single-day dosing and repetitive treatments for 14 or 28 days, produced unequivocal oxidative stress in the form of reduction in the level of the oxidative defense tripeptide GSH in the plasma and brain with a concomitant increase in the oxidative stress biomarker MDA. These antioxidant/oxidative changes induced by statins might have resulted from the metabolic production of reactive oxygen species that subsequently cause organ damage such as the liver at the cellular macromolecular level [20,26]. While the oxidative stress of statins we reported in the present study further supports and ascertains previous studies in which statin therapy was associated with stressful conditions at cellular and vital organ levels [20,21,26], caution should be practiced in interpreting such results. This is because many clinical studies have highlighted the documented beneficial antioxidant effects of statins in practice [22-25]. Nevertheless, in light of our findings using relatively high doses of statins and other experimental findings about the adverse effects of statins reported earlier [8-12,14], we can deduce that oxidative stress is a marker associated with statin overdose and caution should be practiced clinically when treating patients with statins. This notion is especially important as statins can produce liver damage [3] and oxidative stress-related myopathy [27]. Our findings might be important to certain groups of patients such as those with liver failure and renal failure since the kinetics of statins will be different in such patients predisposing them to higher serum concentrations of statins and a higher incidence of side effects like oxidative ones. Therefore, based on our current findings and those of others [20,26], we propose that oxidative stress induced by statins could be associated with liver damage, as the results have shown increased ALT and AST activities, which indicate liver injury.

Additional significant specific organ insult caused by the statins could be the central nervous system since elevated oxidative stress biomarker MDA and the reduction of the antioxidant GSH content were also found in the whole brain of the treated mice in the present study. Albeit, these effects and the reported cholinesterase activity reduction [10,13,14], myopathy [3,5], and the current evidence considering the brain as a non-therapeutic (hypolipidemic) target for statins [28], could be related to neurobehavioral alterations reported in experimental animals [8,14,28]. Keeping these adverse effects in mind, especially those of the single statin doses, and in light of the possibility of statin intolerance [16,17] reported clinically, further in-depth exploration of an animal model for single-dose statin intolerance is warranted. It is, however, difficult to directly extrapolate experimental animal-based data to humans because of species variation, and the drug dosages used clinically could vary considerably from those of the experimental studies in animals, in which doses of statins could be up to 80 times higher than those applied in human beings [1,2,25,29]. This is especially true when the endpoint effect is not the plasma cholesterol [14,28,29].

It should be stressed within the context of potential adverse effects of statins, that such effects could vary among different types of statins since they have non-identical pharmacokinetic and pharmacodynamic properties [1,2,25]. In the present study, atorvastatin (single and repetitive treatments) had the least effect compared to simvastatin and rosuvastatin in producing MDA elevation or GSH reduction in the brain. This outcome could be associated with the reported intrinsic variations in pharmacokinetic properties, together with liver metabolism, neuronal effects, and tissue uptake of a particular statin [2,25,29,30].

Limitations of the study

We used the whole brain for measuring the GSH and MDA levels. However, statin treatments could produce varying changes in GSH and MDA contents among different regions of the brain. In addition, we deduced liver injury from the elevated ALT and AST activities without performing histopathological examinations, which were out of the scope of the current research. It is also possible that statin treatments might have affected other organ systems such as the kidney, as well as other biochemical variables such as the lipid profile of the mice. Albeit, future studies would address these potential research areas.

## Conclusions

The present findings suggest an association of high doses of three statins (atorvastatin, simvastatin, and rosuvastatin) with the induction of oxidative stress, manifested as GSH reduction and MDA elevation, as adverse effects in the brain and plasma of mice, which also suffered from the additional burden of liver injury. These effects could form the basis for an in-depth exploration of the adverse effects of statins in experimental animals and help in identifying a suitable animal model, possibly mice, for the induction of adverse effects of statins that specifically target the brain. This study also highlights the need to further investigate potential statin intolerance outcomes following single-dose treatments in this species. Future studies should aim to examine and delineate any contradictions between the pro-oxidant and antioxidant impacts of statins in experimental animals.

## References

[1] Hirota T, Fujita Y, Ieiri I (2020). An updated review of pharmacokinetic drug interactions and pharmacogenetics of statins. Expert Opin Drug Metab Toxicol.

[2] Climent E, Benaiges D, Pedro-Botet J (2021). Hydrophilic or lipophilic statins?. Front Cardiovasc Med.

[3] Attardo S, Musumeci O, Velardo D, Toscano A (2022). Statins neuromuscular adverse effects. Int J Mol Sci.

[4] Pal S, Sarkar A, Pal PB, Sil PC (2015). Protective effect of arjunolic acid against atorvastatin induced hepatic and renal pathophysiology via MAPK, mitochondria and ER dependent pathways. Biochimie.

[5] Sakaeda T, Kadoyama K, Okuno Y (2011). Statin-associated muscular and renal adverse events: data mining of the public version of the FDA adverse event reporting system. PLoS One.

[6] Darvesh S, Martin E, Walsh R, Rockwood K (2004). Differential effects of lipid-lowering agents on human cholinesterases. Clin Biochem.

[7] Tatley M, Savage R (2007). Psychiatric adverse reactions with statins, fibrates and ezetimibe: implications for the use of lipid-lowering agents. Drug Saf.

[8] Rashid HM, Mohammad FK (2023). Statins modify response of chicks to challenges with xylazine-ketamine and carbaryl. Vet Arhiv.

[9] Cibicková L, Palicka V, Cibicek N, Cermáková E, Micuda S, Bartosová L, Jun D (2007). Differential effects of statins and alendronate on cholinesterases in serum and brain of rats. Physiol Res.

[10] Vukšić A, Lovrić J, Konjevoda P, Blažević N, Bilušić M, Bradamante V (2019). Effects of simvastatin and fenofibrate on butyrylcholinesterase activity in the brain, plasma, and liver of normolipidemic and hyperlipidemic rats. Arh Hig Rada Toksikol.

[11] Hai-Na Z, Xu-Ben Y, Cong-Rong T (2020). Atorvastatin ameliorates depressive behaviors and neuroinflammatory in streptozotocin-induced diabetic mice. Psychopharmacology (Berl).

[12] Bouitbir J, Charles AL, Rasseneur L, Dufour S, Piquard F, Geny B, Zoll J (2011). Atorvastatin treatment reduces exercise capacities in rats: involvement of mitochondrial impairments and oxidative stress. J Appl Physiol (1985).

[13] Rashid HM, Mohammad FK (2023). Differential effects of statins on plasma and brain cholinesterase activities in chicks. Neurosci Res Notes.

[14] Al-Shalchi RF, Mohammad FK Adverse neurobehavioral changes with reduced blood and brain cholinesterase activities in mice treated with statins (In Press). Veterinary World.

[15] Tobert JA (2003). Lovastatin and beyond: the history of the HMG-CoA reductase inhibitors. Nat Rev Drug Discov.

[16] Bytyçi I, Penson PE, Mikhailidis DP (2022). Prevalence of statin intolerance: a meta-analysis. Eur Heart J.

[17] Alonso R, Cuevas A, Cafferata A (2019). Diagnosis and management of statin intolerance. J Atheroscler Thromb.

[18] Deska P, Nowicki M (2017). Short-term changes of serum potassium concentration induced by physical exercise in patient with arterial hypertension treated with angiotensin-converting enzyme inhibitor alone or in combination with statin. J Physiol Pharmacol.

[19] Kwak HB, Thalacker-Mercer A, Anderson EJ (2012). Simvastatin impairs ADP-stimulated respiration and increases mitochondrial oxidative stress in primary human skeletal myotubes. Free Radic Biol Med.

[20] Liu A, Wu Q, Guo J (2019). Statins: adverse reactions, oxidative stress and metabolic interactions. Pharmacol Ther.

[21] Averbukh LD, Turshudzhyan A, Wu DC, Wu GY (2022). Statin-induced liver injury patterns: a clinical review. J Clin Transl Hepatol.

[22] Profumo E, Buttari B, Saso L, Rigano R (2014). Pleiotropic effects of statins in atherosclerotic disease: focus on the antioxidant activity of atorvastatin. Curr Top Med Chem.

[23] Sørensen AL, Hasselbalch HC, Nielsen CH, Poulsen HE, Ellervik C (2019). Statin treatment, oxidative stress and inflammation in a Danish population. Redox Biol.

[24] National Research Council; Division on Earth and Life Studies; Institute for Laboratory Animal Research; Committee for the Update of the Guide for the Care and Use of Laboratory Animals (2011). National Research Council. Guide for the Care and Use of Laboratory Animals. National Research Council. Guide for the Care and Use of Laboratory Animals.

[25] Sirtori CR (2014). The pharmacology of statins. Pharmacol Res.

[26] Pal S, Ghosh M, Ghosh S, Bhattacharyya S, Sil PC (2015). Atorvastatin induced hepatic oxidative stress and apoptotic damage via MAPKs, mitochondria, calpain and caspase12 dependent pathways. Food Chem Toxicol.

[27] Ahmadi Y, Ghorbanihaghjo A, Naghi-Zadeh M, Yagin NL (2018). Oxidative stress as a possible mechanism of statin-induced myopathy. Inflammopharmacology.

[28] Fracassi A, Marangoni M, Rosso P, Pallottini V, Fioramonti M, Siteni S, Segatto M (2019). Statins and the brain: more than lipid lowering agents?. Curr Neuropharmacol.

[29] Kramer AH (2011). Statins in the management of aneurysmal subarachnoid hemorrhage: an overview of animal research, observational studies, randomized controlled trials and meta-analyses. Acta Neurochir Suppl.

[30] Wood WG, Eckert GP, Igbavboa U, Müller WE (2010). Statins and neuroprotection: a prescription to move the field forward. Ann N Y Acad Sci.

